# Draft Genome Sequence of *Dermatophagoides pteronyssinus*, the European House Dust Mite

**DOI:** 10.1128/genomeA.00789-17

**Published:** 2017-08-10

**Authors:** Rose Waldron, Jamie McGowan, Natasha Gordon, Charley McCarthy, E. Bruce Mitchell, Sean Doyle, David A. Fitzpatrick

**Affiliations:** a Department of Biology, Maynooth University, Maynooth, Ireland; b Airmid Healthgroup Ltd., Trinity Enterprise Campus, Dublin, Ireland

## Abstract

*Dermatophagoides pteronyssinus* is the European dust mite and a major source of human allergens. Here, we present the first draft genome sequence of the mite, as well as the *ab initio* gene prediction and functional analyses that will facilitate comparative genomic analyses with other mite species.

## GENOME ANNOUNCEMENT

*Dermatophagoides pteronyssinus*, the European house dust mite, belongs to the family *Pyroglyphidae*, of which more than 6,100 species containing both free-living and parasitic lineages have been described (1). House dust mites live in close association with vertebrates and utilize powerful enzymes to digest organic debris that vertebrates leave behind. Many of these enzymes, secreted in the feces, are major sources of allergens and lead to sensitization in 15 to 20% of the population in industrialized countries, through activation of both innate and adaptive immune responses (2).

For genomic sequencing, we cultured *D. pteronyssinus* (Airmid Healthgroup Ltd., Ireland) for 28 days on house dust mite maximal medium at 75% relative humidity and 25°C. A multi-isolate sample of *D. pteronyssinus* was collected and separated from culture medium by sieving, followed by saturated saline separation. Mites were washed and subjected to 24-h starvation before being sterilized using 70% ethanol; they were then washed and frozen in liquid nitrogen prior to DNA extraction, which was performed using a Promega genomic DNA purification kit and mouse-tail method. DNA was quantified using a Qubit dsDNA BR assay kit and examined for integrity by agarose gel electrophoresis.

Four sequencing libraries—500-bp paired-end (PE), 2-kb mate-pair (MP), 5-kb MP, and 10-kb MP—were prepared for the Illumina HiSeq 2000, 2500, and 4000 platforms (BGI, China) with PE read sizes of 100 bp and MP read sizes of 49 bp. A total of 130,978,913 PE reads, 143,286,220 2-kb MP reads, 56,245,986 5-kb MP reads, and 29,806,232 10-kb MP reads were first trimmed for adapter and base call quality with Trimmomatic (3) before being used for *de novo* assembly in dipSPAdes version 1.0 (4), which resulted in 4,459 contigs with an *N*_50_ of 68,101 bp. Scaffolds were generated using SSPACE (5), and gaps were closed using GapFiller (6). The final assembly resulted in 1,322 scaffolds, with an *N*_50_ value 450,436 bp, an *L*_50_ of 33 scaffolds, and a GC content of 30.93%. The largest scaffold was 3,593,316 bp in length. We estimated the genome size of *D. pteronyssinus* to be approximately 70.76 Mb with a total assembly gap length of 3.14%.

Gene prediction employed AUGUSTUS version 3.1.0 (7), trained using the gene set of *Dermatophagoides farinae* (8). Gene functions were annotated with Pfam domains (9) using InterproScan version 5.3-46.0 (10). CEGMA version 2.5 was used to identify the presence of core eukaryotic protein-coding genes (11). Secreted proteins were predicted using SignalP version 4.1 (12), and transmembrane helices were predicted through the TMHMM server version 2.0 (13).

The *ab initio* gene prediction discovered 12,530 gene models containing 48,371 exons in total. We identified 419 of the 429 CEGMA eukaryotic core genes. We also located full-length sequences for 39 known mite allergens, including the mite group allergens 1 to 11, 13 to 16, 18, and 20 to 33 (8, 14). Functional annotation resulted in gene ontology terms for 5,622 genes and Pfam domains for 8,031 proteins; 1,619 proteins are predicted to have a signal peptide, and 3,610 contain a transmembrane domain.

### Accession number(s).

This whole-genome shotgun project has been deposited at DDBJ/ENA/GenBank under the accession number MQNO00000000. The version described in this paper is the second version, MQNO02000000.

## References

[1] Klimov PB, OConnor B 2013 Is permanent parasitism reversible?—Critical evidence from early evolution of house dust mites. Syst Biol 62:411–423. doi:10.1093/sysbio/syt008. 23417682

[2] Jacquet A 2013 Innate immune responses in house dust mite allergy. ISRN Allergy 2013:735031. doi:10.1155/2013/735031. 23724247 PMC3658386

[3] Bolger AM, Lohse M, Usadel B 2014 Trimmomatic: a flexible trimmer for Illumina sequence data. Bioinformatics 30:2114–2120. doi:10.1093/bioinformatics/btu170. 24695404 PMC4103590

[4] Safonova Y, Bankevich A, Pevzner PA 2014 dipSPAdes: assembler for highly polymorphic diploid genomes. J Comput Biol 22:528–545. doi:10.1089/cmb.2014.0153. PMC4449708 25734602

[5] Boetzer M, Henkel CV, Jansen HJ, Butler D, Pirovano W 2011 Scaffolding pre-assembled contigs using SSPACE. Bioinformatics 27:578–579. doi:10.1093/bioinformatics/btq683. 21149342

[6] Boetzer M, Pirovano W 2012 Toward almost closed genomes with GapFiller. Genome Biol 13:R56. doi:10.1186/gb-2012-13-6-r56. 22731987 PMC3446322

[7] Stanke M, Diekhans M, Baertsch R, Haussler D 2008 Using native and syntenically mapped cDNA alignments to improve *de novo* gene finding. Bioinformatics 24:637–644. doi:10.1093/bioinformatics/btn013. 18218656

[8] Chan TF, Ji KM, Yim AKY, Liu XY, Zhou JW, Li RQ, Yang KY, Li J, Li M, Law PTW, Wu YL, Cai ZL, Qin H, Bao Y, Leung RKK, Ng PKS, Zou J, Zhong XJ, Ran PX, Zhong NS, Liu ZG, Tsui SKW 2015 The draft genome, transcriptome, and microbiome of *Dermatophagoides farinae* reveal a broad spectrum of dust mite allergens. J Allergy Clin Immunol 135:539–548. doi:10.1016/j.jaci.2014.09.031. 25445830

[9] Finn RD, Coggill P, Eberhardt RY, Eddy SR, Mistry J, Mitchell AL, Potter SC, Punta M, Qureshi M, Sangrador-Vegas A, Salazar GA, Tate J, Bateman A 2016 The Pfam protein families database: towards a more sustainable future. Nucleic Acids Res 44:D279–DD285. doi:10.1093/nar/gkv1344. 26673716 PMC4702930

[10] Jones P, Binns D, Chang HY, Fraser M, Li W, McAnulla C, McWilliam H, Maslen J, Mitchell A, Nuka G, Pesseat S, Quinn AF, Sangrador-Vegas A, Scheremetjew M, Yong SY, Lopez R, Hunter S 2014 InterProScan 5: genome-scale protein function classification. Bioinformatics 30:1236–1240. doi:10.1093/bioinformatics/btu031. 24451626 PMC3998142

[11] Parra G, Bradnam K, Korf I 2007 CEGMA: a pipeline to accurately annotate core genes in eukaryotic genomes. Bioinformatics 23:1061–1067. doi:10.1093/bioinformatics/btm071. 17332020

[12] Petersen TN, Brunak S, von Heijne G, Nielsen H 2011 SignalP 4.0: discriminating signal peptides from transmembrane regions. Nat Methods 8:785–786. doi:10.1038/nmeth.1701. 21959131

[13] Mulay SR, Desai J, Kumar SV, Eberhard JN, Thomasova D, Romoli S, Grigorescu M, Kulkarni OP, Popper B, Vielhauer V, Zuchtriegel G, Reichel C, Bräsen JH, Romagnani P, Bilyy R, Munoz LE, Herrmann M, Liapis H, Krautwald S, Linkermann A, Anders HJ 2016 Cytotoxicity of crystals involves RIPK3-MLKL-mediated necroptosis. Nat Commun 7:10274. doi:10.1038/ncomms10274. 26817517 PMC4738349

[14] Rider SD, Morgan MS, Arlian LG 2015 Draft genome of the scabies mite. Parasit Vectors 8:585. doi:10.1186/s13071-015-1198-2. 26555130 PMC4641413

